# Growth and Nitrogen Uptake Kinetics in Cultured *Prorocentrum donghaiense*

**DOI:** 10.1371/journal.pone.0094030

**Published:** 2014-04-07

**Authors:** Zhangxi Hu, Shunshan Duan, Ning Xu, Margaret R. Mulholland

**Affiliations:** 1 Key Laboratory of Marine Ecology and Environmental Sciences, Institute of Oceanology, Chinese Academy of Sciences, Qingdao, China; 2 Research Center of Hydrobiology, Jinan University, Guangzhou, Guangdong, China; 3 Department of Ocean, Earth and Atmospheric Sciences, Old Dominion University, Norfolk, Virginia, United States of America; University of Connecticut, United States of America

## Abstract

We compared growth kinetics of *Prorocentrum donghaiense* cultures on different nitrogen (N) compounds including nitrate (NO_3_^−^), ammonium (NH_4_^+^), urea, glutamic acid (glu), dialanine (diala) and cyanate. *P. donghaiense* exhibited standard Monod-type growth kinetics over a range of N concentraions (0.5–500 μmol N L^−1^ for NO_3_^−^ and NH_4_^+^, 0.5–50 μmol N L^−1^ for urea, 0.5–100 μmol N L^−1^ for glu and cyanate, and 0.5–200 μmol N L^−1^ for diala) for all of the N compounds tested. Cultures grown on glu and urea had the highest maximum growth rates (μ_m_, 1.51±0.06 d^−1^ and 1.50±0.05 d^−1^, respectively). However, cultures grown on cyanate, NO_3_^−^, and NH_4_^+^ had lower half saturation constants (K_μ_, 0.28–0.51 μmol N L^−1^). N uptake kinetics were measured in NO_3_^−^-deplete and -replete batch cultures of *P. donghaiense*. In NO_3_^−^-deplete batch cultures, *P. donghaiense* exhibited Michaelis-Menten type uptake kinetics for NO_3_^−^, NH_4_^+^, urea and algal amino acids; uptake was saturated at or below 50 μmol N L^−1^. In NO_3_^−^-replete batch cultures, NH_4_^+^, urea, and algal amino acid uptake kinetics were similar to those measured in NO_3_^−^-deplete batch cultures. Together, our results demonstrate that *P. donghaiense* can grow well on a variety of N sources, and exhibits similar uptake kinetics under both nutrient replete and deplete conditions. This may be an important factor facilitating their growth during bloom initiation and development in N-enriched estuaries where many algae compete for bioavailable N and the nutrient environment changes as a result of algal growth.

## Introduction

Harmful algal blooms (HABs) have increased in coastal waters worldwide [1]–[4], exerting serious economic impacts on marine fisheries and aquaculture, and threatening public health and aquatic ecosystems [1], [5]. In Chinese coastal waters, HABs have increased in frequency, intensity, and duration in recent decades [6]–[8]. *Prorocentrum donghaiense* is a HAB species that frequently blooms during late spring and early summer in coastal waters around the East China Sea, including the Changjiang River Estuary, and coastal waters adjacent to Zhejiang in China [9]–[11], and coastal areas near Japan and South Korea [9]. Between 2000 and 2006, about 120 *P. donghaiense* bloom events (maximum cell densities of up to 3.6×10^8^ cells·L^−1^) were reported from Chinese coastal waters (data from State Oceanic Administration, China) [2] and the spatial extent of these blooms ranged from several thousand to more than ten thousand km^2^. Blooms persisted several days to a month, during which time they caused serious economic impacts to marine fisheries, public health, and aquatic ecosystems [8], [10], [11]. Despite documentation regarding their impacts, the environmental factors promoting *P. donghaiense* bloom initiation and persistence are still unclear.

HABs are often linked to nutrient over-enrichment and consequent eutrophication of coastal waters including the East China Sea [5], [12]–[14]. The Changjiang River Estuary and East China Sea, where *P. donghaiense* blooms frequently occur, are hydrologically complex; they receive terrestrial nutrient inputs and freshwater through the watershed, but are also influenced by oceanic circulation that can affect particle transport in the estuary [15]. High nutrient loads to the watershed have been implicated as the proximate cause of *P. donghaiense* blooms in this system [16].

Dissolved inorganic N concentrations in the Changjiang River Estuary, mainly in the form of NO_3_^−^, increased several-fold in the past few decades, reaching as high as 131.6 μmol N L^−1^ [15]–[18]. However, field studies have shown that NO_3_^−^ is rapidly consumed during the initiation of *P. donghaiense* blooms leading to NO_3_^−^-depletion during blooms [16]. Our previous culture study determined that *P. donghaiense* can grow on multiple forms of N when they are supplied in saturating concentrations (50 μmol N L^−1^) [19]. However, it is not known whether *P. donghaiense* has a strong affinity for NO_3_^−^ or sources of recycled N (e.g., NH_4_^+^, urea, and amino acids) when they are present at lower concentrations, more realistic of environmental concentrations once blooms are established.

The dissolved N pool in aquatic systems is dynamic, consisting of varying concentrations of dissolved inorganic N (DIN) and dissolved organic N (DON) compounds, many of which remain to be identified [20]–[22]. In Chinese coastal waters DIN (NO_3_^−^+NO_2_^−^+NH_4_^+^) concentrations ranged 0–45.0 μmol N L^−1^ in the surface waters of Southern Yellow Sea during 2006–2007 [23] and 0–60.0 μmol N L^−1^ in waters adjacent to Hong Kong [24]. In the Changjiang River estuary and East China Sea, DIN (NO_3_^−^, NO_2_^−^ and NH_4_^+^) concentrations ranged 0–131.6 μmol N L^−1^ [15]–[18] over the same time period. In addition to DIN, urea can be an important component of the dissolved N pool and accounted for ∼10–60% of the total dissolved N in Hong Kong waters in summer of 2008 [24]. In the East China Sea, DON concentrations ranged 5.78–25.26 μmol N L^−1^, accounting for 44.0–88.5% of total dissolved nitrogen [16]. While concentrations of urea and DFAA were only a small component of the DON pool, 0.21–1.39 and 0.09–1.55 μmol N L^−1^, respectively, these compounds are labile and rapidly regenerated and turned over in the environment [21], [22].

To date, our understanding of the nitrogenous nutrition of *P. donghaiense* is limited. While previous research demonstrated that *P. donghaiens*e can potentially utilize multiple inorganic and organic N compounds to grow [19], little is known about their affinity and capacity for growth on the diverse range of N compounds found in the environment. Therefore, we compared growth kinetics of *P. donghaiense* on medium supplied with NO_3_^−^, NH_4_^+^, urea, glu, dialanine (diala) or cyanate as the sole source of N. In addition, we used stable isotopes as tracers to compare uptake kinetics for these compounds in cultures acclimated under NO_3_^−^-replete and -deplete culture conditions, conditions likely present in the environment during bloom initiation and maintenance phases, respectively.

## Materials and Methods

### 2.1 Culture conditions

*Prorocentrum donghaiense*, was isolated from the East China Sea, and obtained from the Research Center of Hydrobiology, Jinan University, Guangzhou, China. Cultures were grown in artificial seawater enriched with, sterile, silicate-free f/2 trace metals and vitamins [25]. Concentrations of NO_3_^−^ were adjusted to conform to different experimental treatments (see below). Cultures were maintained in an environmental room at constant temperature (23±0.5°C) and irradiance (60±5 μmol quanta m^−2^ s^−1^) on a 12 h∶12 h (light: dark) light cycle and transferred to fresh medium every week to ensure cells remained in exponential growth phase for the duration of the experiments.

For all experiments, dissolved nutrients were measured after filtering through a 0.2 μm Supor filter. Samples were stored frozen until analysis. NO_3_^−^ and urea were measured on an Astoria Pacific autoanalyzer according to the manufacturer's specifications and using colorimetric methods [26]. NH_4_^+^ was determined using the manual phenol hypochlorite method [27]. Concentrations of dissolved free amino acids (DFAA) were measured by high performance liquid chromatography (HPLC) [28]. Cells were enumerated using a FACSCalibur flow cytometer (Becton Dickinson Instruments). Samples were also collected onto GF/F filters and frozen for analysis of chlorophyll *a* (Chl *a*). Chl *a* concentrations were measured fluorometrically within one week of sample collection after extraction of cells in 90% acetone [29].

### 2.2 Experimental design

#### 2.2.1 Growth kinetics experiments

Growth kinetics of *P. donghaiense* were determined for six different N compounds. Cultures were grown in triplicate, capped, 50 ml Pyrex test tubes containing 35 ml of culture suspended in f/2 modified medium amended with 0.5, 2, 5, 20, 50, 100, 200, or 500 μmol N L^−1^ supplied in the form of NO_3_^−^, NH_4_^+^, urea, glu, diala, or cyanate. Cultures were maintained in the environmental chamber under conditions described above for at least three generations prior to experiments in order to ensure cells were acclimated to treatment conditions. *In vivo* fluorescence was monitored to estimate chlorophyll biomass using a Turner Designs AU-10 fluorometer at the same time each day. Growth rates, *μ* (day^−1^), were calculated using a least squares fit to a straight line after logarithmic transformation of *in vivo* fluorescence data as described by Guillard [30] using the equation:
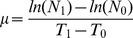
(1)where N_1_ and N_0_ were the biomass (*in vivo* fluorescence) at time T_1_ and T_0_, respectively, during the linear portion of exponential phase growth.

The relationship between specific growth rate and N concentration was fitted to a Monod growth kinetic model using the equation:
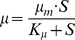
(2)where *μ* was the specific growth rate (day^−1^) calculated during the linear portion of exponential phase growth (Equation 1), *μ_m_* was the maximum specific growth rate (day^−1^), S was the N concentration (μmol N L^−1^), and K_μ_ (half saturation constant; μmol N L^−1^) was the N concentration at μ_m_/2 [31].

#### 2.2.2 N uptake kinetic experiments

For N uptake kinetic experiments, cultures of *P. donghaiense* were grown on f/2 medium modified to contain 50 μmol N L^−1^ as NO_3_^−^ in two, 5-liter bottles. Cell abundance and ambient NO_3_^−^ concentrations were monitored daily. N-deplete uptake kinetic experiments were initiated after NO_3_^−^ concentrations were below the detection limit (0.05 μmol N L^−1^) for 5 consecutive days. Uptake kinetics experiments were initiated by dispensing 35 ml of NO_3_^−^-deplete culture into 50 ml Pyrex test tubes and adding ^15^NO_3_^−^, ^15^NH_4_^+^, ^15^N-^13^C dually labeled urea, glu, or an algal amino acid mixture to duplicate test tubes at final substrate concentrations of 0.1, 0.2, 0.5, 1.0, 2.0, 5.0, 10.0, 20.0 and 50.0 μmol N L^−1^. For NO_3_^−^-replete uptake kinetic experiments, 35 ml of culture was dispensed into a series of 50 ml Pyrex test tubes and each was amended with an additional 50.0 μmol N L^−1^ as ^14^NO_3_^−^ to ensure the cultures were NO_3_^−^-replete. As for NO_3_^−^-replete treatments, ^15^NH_4_^+^, and ^15^N-^13^C dually labeled urea, glu, or an algal amino acid mixture were added to duplicate tubes at the same concentrations indicated above, however, NO_3_^−^ uptake kinetics could not be measured in these NO_3_^−^-replete cultures.

Uptake experiments were incubated at ∼23°C and at an irradiance of ∼60 μmol quanta m^−2^ s^−1^. After <1 hour, incubations were terminated by gentle filtration through precombusted (450°C for 2 hours) GF/F (nominal pore size ∼0.7 μm) filters. Filters were immediately frozen in sterile polypropylene cryovials until analysis. Frozen samples were dried in an oven at 40°C for 2 days and pelletized into tin discs prior to analysis on a Europa Scientific 20–20 isotope ratio mass spectrometer equipped with an automated N and C analyzer preparation unit. Specific uptake rates were calculated using a mixing model [32]:
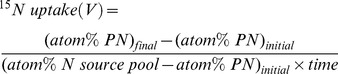
(3)where the source pool was the dissolved N pool that was enriched during uptake experiments. Specific uptake rates (V; h^−1^) at each N concentration for each N substrate tested were fitted to a Michaelis-Menten model using the following equation:
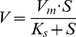
(4)where V_m_ is the maximum specific uptake rate (h^−1^); S the substrate concentration (μmol N L^−1^); and K_s_ the half saturation constant, the concentration (μmol N L^−1^) where V is equivalent to half of V_m_, for the N substrate tested.

### 2.3 Statistical analysis

All statistical tests were carried out using Origin 7.0 and Microsoft Excel 2007 with the level of significance set at an α equal to 0.05. Differences between growth and uptake kinetic parameters were compared using a one-way ANOVA test. Monod and Michaelis-Menten curves were fitted using Origin 7.0.

## Results

### 3.1 Growth kinetics of *P. donghaiense*

**Figure 1:**
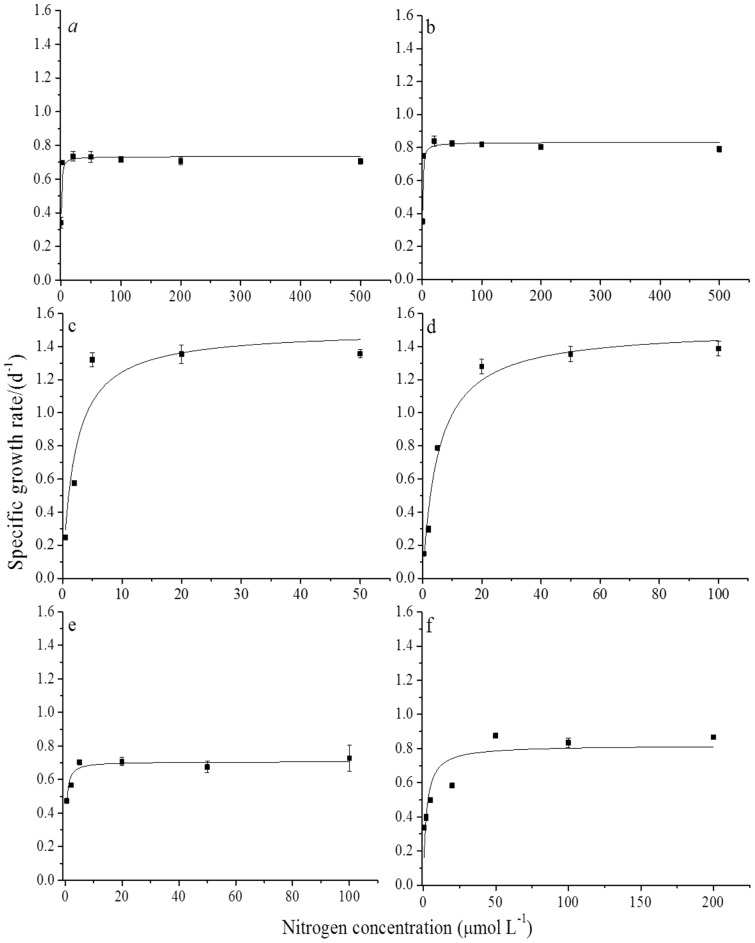
Growth kinetics for *P*. *donghaiense*. Growth rate as a function of NO_3_^−^ (a), NH_4_^+^ (b), urea (c), glu (d), cyanate (e) and diala (f) concentrations in batch cultures of *P. donghaiense*. Solid lines were fitted iteratively to the data according to the Monod equation and kinetic parameters were calculated as described in the text.

*P. donghaiense* exhibited standard Monod-type growth kinetics over N concentraions ranging from 0.5–500 μmol N L^−1^ for NO_3_^−^ and NH_4_^+^, 0.5–50 μmol N L^−1^ for urea, 0.5–100 μmol N L^−1^ for glu and cyanate, and 0.5–200 μmol N L^−1^ for diala (Fig. 1). Maximum specific growth rates (μ_m_) ranged from 0.71±0.04 to 1.51±0.06 d^−1^ (Table 1). The highest maximum growth rates were observed in cultures growing with glu or urea as the sole N source; maximum growth rates were a factor of 2 lower in cultures grown with NO_3_^−^, NH_4_^+^, cyanate, or diala supplied as the sole N source (Table 1). Half saturation constants (K*_μ_*) ranged from 0.28±0.08 to 5.25±0.40 μmol N L^−1^. The lowest K*_μ_* concentrations were observed in *P. donghaiense* cultures growing on media containing NO_3_^−^, NH_4_^+^, and cyanate as the sole source of N (0.28–0.51 μmol N L^−1^) (Table 1). In contrast, K*_μ_* concentrations were an order of magnitude higher in cultures grown with urea, glu, or diala as the sole source of N suggesting higher affinities for cyanate, NO_3_^−^, NH_4_^+^, and urea than for glu and diala (Table 1).

**Table 1:** Mean(±SD, n = 3) growth kinetic parameters for *P. donghaiense* growing with NO_3_^−^, NH_4_^+^, urea, glu, cyanate and diala as the sole source of N in the culture medium.

Nitrogen species	μ_m_ (d^−1^)	K_μ_ (μmol N L^−1^)	Affinity (α) (μ_m_/K_μ_)	R^2^
NO_3_^−^	0.74±0.02	0.42±0.02	1.74±0.13	0.82
NH_4_^+^	0.83±0.02	0.51±0.03	1.65±0.10	0.92
Urea	1.50±0.05	2.05±0.04	0.73±0.02	0.91
Glu	1.51±0.06	5.25±0.40	0.29±0.01	0.98
cyanate	0.71±0.04	0.28±0.08	2.58±0.49	0.85
Diala	0.82±0.01	2.05±0.02	0.40±0.01	0.76

The maximum specific growth rate (μ_m_), half saturation constant (K_μ_) and affinity (α) for each nitrogen compound was calculated according to the Monod model.

### 3.2 N uptake kinetics by *P. donghaiense*

**Figure 2:**
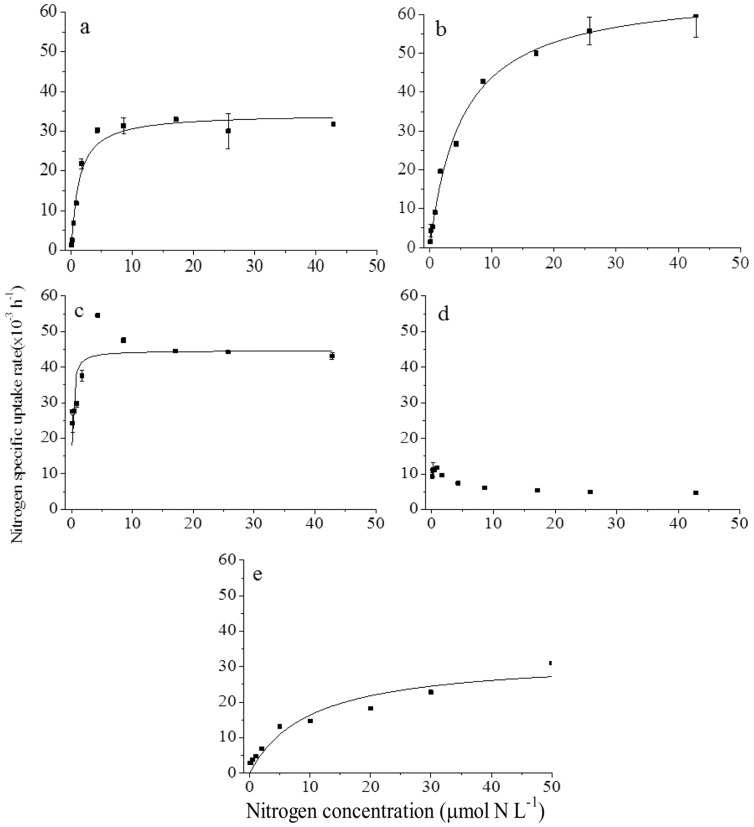
N uptake kinetics for *P*. *donghaiense* growing in NO_3_^−^-deplete batch cultures. Nitrogen uptake as a function of NO_3_^−^ (a), NH_4_^+^ (b), urea (c), glu (d), and algal amino acids (e) concentration in batch cultures of *P. donghaiense* growing on NO_3_^−^-deplete media. Solid lines were fitted iteratively to the data according to the Michaelis-Menten equation and kinetic parameters were calculated as described in the text.

**Figure 3:**
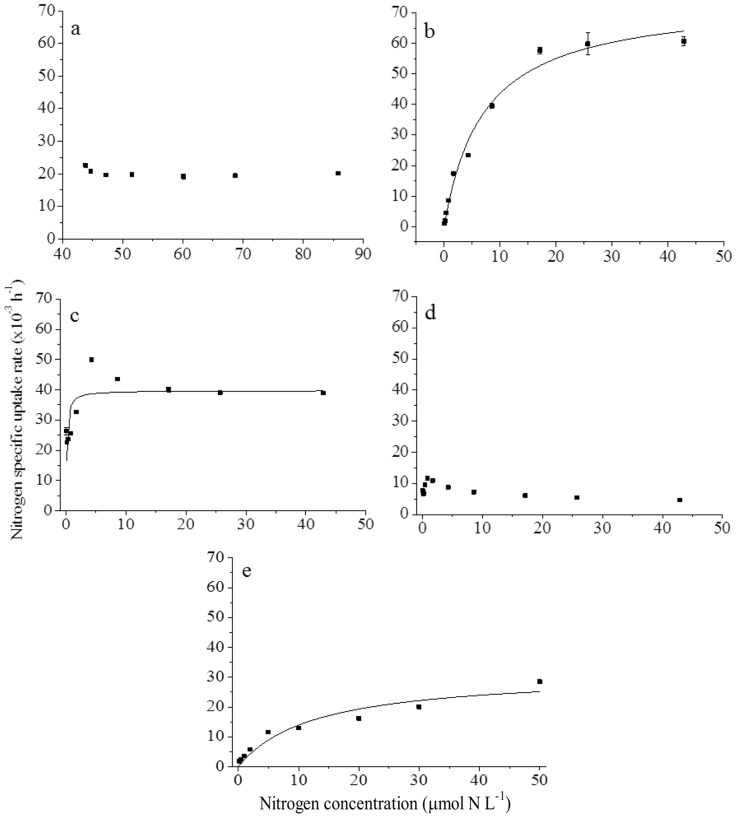
N uptake kinetics for *P*. *donghaiense* growing in NO_3_^−^-replete batch cultures. Nitrogen uptake rates as a function of NO_3_^−^ (a), NH_4_^+^ (b), urea (c), glu (d), and algal amino acid (e) concentrations in batch cultures of *P. donghaiense* growing on NO_3_^−^-replete media. Solid lines were fitted iteratively to the data according to the Michaelis-Menten equation and kinetic parameters were calculated as described in the text.

Kinetics for NH_4_^+^, urea, and algal amino acids conformed to Michaelis-Menten kinetics in both NO_3_-deplete and -replete cultures of *P. donghaiense* (Table 2; Figs. 2 and 3). While NO_3_^−^ uptake also conformed to Michaelis-Menten kinetics in NO_3_-deplete cultures (Fig. 2a), NO_3_^−^ uptake kinetics could not be measured in NO_3_^−^-replete cultures because of the excess NO_3_^−^ in the growth media (Fig. 3a). Glu uptake did not conform to the Michaelis-Menten kinetic model in either NO_3_-deplete (Fig. 2d) or -replete (Fig. 3d) cultures of *P. donghaiense* and higher glu uptake rates were observed at lower glu concentrations.

**Table 2:** Mean (±SD, n = 2) uptake kinetic parameters (maximum specific uptake rate (V_m_), half saturation constant (K_s_) and affinity (α)) for *P*. *donghaiense* growing on NO_3_^−^ deplete culture media.

Nitrogen species	V_m_ (×10^−3^ h^−1^)	K_s_ (μmol N L^−1^)	Affinity (α) (V_m_/K_s_)	R^2^
NO_3_^−^ deplete cultures:				
NO_3_^−^	34.4±0.9	1.3±0.1	27.7±2.4	0.97
NH_4_^+^	66.9±6.0	5.3±1.1	12.8±1.6	0.99
Urea	44.7±0.4	0.13±0.01	339.4±26.3	0.61
algal amino acids	32.6±1.0	9.9±0.9	3.3±0.2	0.93
NO_3_^−^ replete cultures (50 μmol L^−1^ NO_3_^−^):				
NH_4_^+^	74.7±3.4	7.1±0.4	10.5±0.1	0.99
Urea	39.7±0.2	0.12±0.01	332.9±26.7	0.49
algal amino acids	31.4±0.4	12.5±0.1	2.5±0.0	0.94

Results from glu uptake kinetic studies did not conform to Michaelis-Menten kinetics so kinetic parameters (Figs. 2d and 3d) so are not included here.

Uptake kinetics for NO_3_^−^, NH_4_^+^, urea, and algal amino acids followed Michaelis-Menten type uptake kinetics in NO_3_^−^-deplete batch cultures of *P. donghaiense* (Table 2; Fig. 2). Although results were sufficient to fit a Michaelis-Menten model for NH_4_^+^ uptake, NH_4_^+^ uptake by NO_3_^−^-deplete *P. donghaiense* was not saturated even at the highest concentrations used in this experiment (Fig. 2b). Maximum specific uptake rates (V_m_) of (66.9±6.0)×10^−3^ h^−1^ were calculated for NH_4_^+^ (Table 2), while V_m_ calculated for NO_3_^−^ ((34.4±0.9)×10^−3^ h^−1^), urea ((44.7±0.4)×10^−3^ h^−1^), and the algal amino acid mixture ((32.6±1.0)×10^−3^ h^−1^) were lower (Table 2). The half saturation constants (K_s_) for uptake of NO_3_^−^, NH_4_^+^, urea, and algal amino acids by NO_3_^−^-deplete *P. donghaiense* were 1.3±0.1, 5.3±1.1, 0.13±0.01 and 9.9±0.9 μmol N L^−1^, respectively (Table 2). The α values, which are often used as an index of nutrient affinity, were 27.7±2.4, 12.8±1.6, 339.4±26.3 and 3.3±0.2 for NO_3_^−^, NH_4_^+^, urea, and algal amino acids, respectively (Table 2) suggesting NO_3_^−^-deplete *P. donghaiense* had the highest affinity for urea and the lowest for amino acids.

In NO_3_^−^-replete cultures of *P. donghaiense*, maximum specific uptake rates (V_m_) of NH_4_^+^ were greatest and not significantly different than those observed in NO_3_^−^-deplete cultures (*p*>0.05; Table 2) (Table 2). Similarly, V_m_ for urea and algal amino acids were lower than those observed for NH_4_^+^ but comparable to those observed in NO_3_^−^-deplete cultures (Table 2; Figs. 2 and 3). For urea, the V_m_ was significantly higher (*p*<0.05) but for algal amino acids there was no significant difference (*p*>0.05) between NO_3_^−^-replete and NO_3_^−^- deplete cultures of *P. donghaiense* (Table 2). Although there were significant differences between K_s_ values for NH_4_^+^ and algal amino acid uptake (*p*<0.05) between NO_3_^−^-replete and -deplete cultures of *P. donghaiense*, the K_s_ values for NH_4_^+^, urea, and the algal amino acid uptake in NO_3_^−^-replete cultures of *P. donghaiense* were 7.1±0.4, 0.1±0.01 and 12.5±0.1 μmol N L^−1^ h^−1^, respectively (Table 2), similar to and showing the same pattern as values measured in NO_3_^−^-deplete cultures. Similarly, the derived α values, a measure of nutrient affinity, were also similar for NO_3_^−^-replete and -deplete cultures of *P. donghaiense* even though there were significant differences (*p*<0.05) in α between NO_3_^−^-replete and -deplete cultures for algal amino acids (Table 2).

## Discussion

Nutrient enrichment has been implicated as a causal factor in the occurrence of HAB events worldwide [5], [14], [33]. Both the form of dissolved N and its concentration in coastal and marine environments are thought to be important to the formation and development of HABs and phytoplankton blooms in general [13], [15], [34], [35]. However, different phytoplankton species and groups have different uptake capacities and affinities for specific inorganic and organic nitrogen compounds [19], [20], [36]. Many phytoplankton can also mobilize N from complex organic compounds such as peptides using extracellular enzymes thereby making N from these compounds available for uptake [32], [37]. In coastal areas, the concentration and composition of the dissolved N pool and the relative concentrations of various N compounds vary temporally and spatially, likely impacting the phytoplankton community composition and abundance on both short and longer timescales [15], [38], [39].

*P. donghaiense* is a mixotrophic species capable of both autotrophic and heterotrophic metabolisms. Previous research demonstrated that *P. donghaiense* can utilize inorganic and organic N compounds including urea, amino acids, small peptides, and cyanate [16], [19] as N sources. In addition, this organism can also ingest cyanobacteria, cryptophytes, and other dinoflagellates [40]. To better understand the N nutrition and capabilities of *P. donghaiense*, we conducted growth and N uptake kinetic experiments in cultured isolates to determine their affinity, preference, and capacity for growth on different N compounds and found that *P. donghaiense* had high maximum growth rates and exhibited high uptake capacities and affinities for a diverse suite of N compounds under both NO_3_^−^ -deplete and -replete conditions.

### 4.1 Growth kinetics

The Monod equation [41] has been used to describe saturation kinetics for nutrient-limited phytoplankton growth in many field and laboratory studies [42]–[44]. Maximum specific growth rates (μ_m_) and half saturation constants (K_μ_) are two important kinetic parameters derived from this model that quantify organismal growth responses to environmental nutrient concentrations. The initial slope of the Monod function (μ_m_/K_μ_) is thought to be a competitive index and used to assess the affinity of phytoplankton for particular nutrient substrates [45]. This model provides several useful tools for evaluating fitness of co-occurring phytoplankton with respect to the external nutrient availability in the environment [46]. Theoretical studies of resource competition suggest that the functional relationship between growth rate and the concentration of nutrient elements in the environment determines a species' ability to compete for that nutrient in the environment [46].

In our culture experiments, we found *P. donghaiense* grew well on DIN (NO_3_^−^ and NH_4_^+^) and DON (urea, glu, diala and cyanate) compounds when they were supplied as the sole source of N (Fig. 1). *P. donghaiense* had comparable maximum specific growth rates when growing on NO_3_^−^, NH_4_^+^, cyanate, or diala (0.71–0.83 d^−1^); but maximum specific growth rates were a factor of 2 higher in cultures supplied with urea or glu as the sole source of N (1.50 and 1.51 d^−1^, respectively; Table 1). In contrast, half saturation constants for growth (K_μ_) were more variable (0.28±0.08 to 5.25±0.40 μmol N L^−1^); K_μ_ values were lower (0.28–0.51 μmol N L^−1^) for cultures growing on NO_3_^−^, NH_4_^+^, or cyanate, and were an order of magnitude higher (2.05–5.25 μmol N L^−1^) in cultures grown on urea, glu, or diala (Table 1), suggesting a higher affinity for the inorganic N compounds. So, while *P. donghaiense* had higher maximum growth rates when supplied urea or glu as a sole source of N, cells had a higher affinity for cyanate, NO_3_^−^, and NH_4_^+^ relative to urea, glu, and diala (Table 1) suggesting that the former compounds might be important sources N when nutrient concentrations are submicromolar.

Calculated growth kinetic parameters for *P. donghaiense* indicate that maximum specific growth rates and half saturation constants (K_μ_) for NO_3_^−^, NH_4_^+^, and urea, were comparable to each other and those measured for other bloom-forming dinoflagellate species, *Prorocentrum minimum*, *Cochlodinium polykrikoides* and for *Scrippsiella trochoidea* growing on urea (Table 3). Maximum growth rates for *P. donghaiense* growing with NO_3_^−^, NH_4_^+^, urea or glu as the sole source of N were higher than those previously measured for *P. minimum*, *C. polykrikoides*, and *S. trochoidea* growing on the same N compounds (Table 3). *S*. *trochoidea*, *P. minimum* and *C. polykrikoides* had comparable maximum specific growth rates when going on urea (0.44–0.47 d^−1^; Table 3), but these were all lower than the maximum specific growth rate observed for *P. donghaiense* in this study(1.50 d^−1^) (Table 1). While *P. donghaiense* (this study) and *C. polykrikoides* [44] exhibited comparable maximum specific growth rates on NO_3_^−^, NH_4_^+^, urea and glu, *P. minimum* exhibited higher specific growth rates in cultures growing on urea or NO_3_^−^ (0.45 and 0.43±0.01 d^−1^, respectively) relative to cultures growing on NH_4_^+^ or glu (0.27±0.05 and 0.23±0.02 d^−1^, respectively) [43].

**Table 3:** Summary of mean (±SD) maximum specific growth rates (μ_max_, d^−1^) and half saturation constants (k_μ_, μmol N L^−1^) for nitrate (NO_3_^−^), ammonium (NH_4_^+^), urea, and glutamic acid (glu) estimated for different dinoflagellate species.

Species	NO_3_^−^		NH_4_^+^		Urea		glu		Reference
	μ_max_	k_μ_	μ_max_	k_μ_	μ_max_	k_μ_	μ_max_	k_μ_	
*Prorocentrum minimum*	0.43±0.01	1.10±0.40	0.23±0.02	0.70±0.40	0.45	<0.50	0.27±0.05	53.0±31.0	[43]
*Cochlodinium polykrikoides*	0.43±0.01	2.06±0.32	0.44±0.02	2.69±0.49	0.44±0.01	2.90±0.46	0.53±0.02	1.91±0.47	[44]
*Scrippsiella trochoidea*	-	-	-	-	0.47	21.83	-	-	[47]
*P. donghaiense*	0.74±0.02	0.42±0.02	0.83±0.02	0.51±0.03	1.50±0.05	2.05±0.04	1.51±0.06	5.25±0.40	This study

But unlike *P. donghaiense*, *P*. *minimum* had the lowest half saturation constants for growth on urea, followed by NO_3_^−^ and NH_4_^+^; both had the highest half saturation constants for glu (Table 3) [43]. In contrast, *C. polykrikoides* had higher half saturation constants for urea and NH_4_^+^ than for NO_3_^−^ and glu [44]. The half saturation constant for *S. trochoidea* growing on urea was much higher than for the other three dinoflagellates suggesting a low affinity for growth on this compound [47].

In this study, *P. donghaiense* also exhibited maximum growth rates and half saturation constants comparable to those for growth on NH_4_^+^ and NO_3_^−^, when growing with cyanate and dialanine as the sole source of N (Fig. 1; Table 1). Because peptides and cyanate are degradation products of decaying cells [32], [48], these compounds were likely available to *P. donghaiense* during bloom maintenance when NO_3_^−^ concentrations are exhausted and recycling processes are important for maintaining cell biomass and turnover. The concentration and turnover of these organic compounds have not been assessed in natural systems where blooms of *P. donghaiense* occur. Comparisons of *P. donghaiense* growth kinetics with those of co-occurring species relative to the bioavailable DIN and DON compounds in the environment is warranted to elucidate how competitive interactions among phytoplankton populations contributes to bloom initiation and maintenance.

### 4.2 Uptake kinetics

The Michaelis-Menten equation is often used to describe the relationship between external nutrient concentrations and their uptake rates [49]–[51]. As for growth kinetics, kinetic parameters for N uptake have been used to assess the relative preference (and affinity) for different N substrates in the environment [50], [52]. While kinetic parameters are usually measured in nutrient-deplete cultures, we conducted N uptake kinetic experiments in both NO_3_^−^-deplete and -replete batch cultures of *P. donghaiense* because NO_3_^−^ concentrations are generally high in the Changjiang River estuary when blooms of this organism initiate [15]–[18] but then become rapidly depleted as algal biomass increases. Here we compared N uptake kinetics for NO_3_^−^ (N-deplete cultures only), NH_4_^+^, urea, and algal amino acids in N-replete and –deplete cultures of *P. donghaiense* (Table 2; Figs. 2, 3).

Michaelis-Menten type uptake kinetics were observed for all N compounds tested except glu (Figs. 2d, 3d). For NH_4_^+^, the uptake capacity (V_m_) for exponentially growing *P. donghaiense* was ∼1.5, 1.9, and 2.1 times greater than those for urea, NO_3_^−^, and the algal amino acids, respectively, in both NO_3_^−^-deplete and -replete cultures. The half saturation constants, which are also used to estimate nutrient affinity under nutrient-limiting conditions, suggest similar nutrient preferences under NO_3_^−^-deplete and –replete conditions; urea >>>NO_3_^−^>NH_4_^+^> algal amino acids for NO_3_^−^-deplete *P. donghaiense*, and urea >>>NH_4_^+^> algal amino acids for NO_3_^−^-replete *P. donghaiense* (Table 2; Figs. 2, 3).

Differences in kinetic parameters are often observed in cells growing under N-replete versus -deplete conditions [53]. Many N-starved cells are capable of enhanced N uptake when nutrients are resupplied, and N-replete cells can have higher half-saturation constants than those grown under N-depleted conditions [36]. While our kinetics parameters conformed to this norm based on our statistical analyses, it was surprising that uptake kinetics for NH_4_^+^, urea, and amino acids were similar in nutrient-replete and -deplete cultures of *P. donghaiense* (Table 2; Figs. 2&3). Ambient NO_3_^−^ concentrations were ∼43 μmol N L^−1^ when we initiated our uptake experiments in the N-replete cultures and this should have been saturating for NO_3_^−^ uptake (Table 2). However, kinetic parameters in NO_3_^−^-replete and -deplete batch cultures were similar suggesting that even when NO_3_^−^ concentrations are high, such as during bloom initiation, *P. donghaiense* has a high affinity for other N compounds that may available in the environment and this may give them a competitive edge over other phytoplankton.

NO_3_^−^ and NH_4_^+^ are the dominant DIN species thought to fuel the bulk of primary production in estuarine, coastal, and oceanic environments. In Chinese estuaries and coastal waters where *P. donghaiense* blooms, NO_3_^−^ and NH_4_^+^ concentrations are commonly 0–20.0 and 0–3.0 μmol N L^−1^, respectively [16]. In this study, we found *P. donghaiense* had higher maximum uptake rates for NH_4_^+^ than NO_3_^−^, urea and amino acids in both NO_3_^−^-deplete and -replete batch cultures (Table 2; Fig. 2). However, because of the low half saturation constants for urea uptake (0.13 and 0.12, respectively), both N-deplete and -replete cultures of *P. donghaiense* had a much higher affinity for urea than for inorganic N or amino acids. This is consistent with results from a field study in the East China Sea during a *P. donghaiense* dominant bloom [11], [16]. As we know, DON can constitute more than 80% of the TDN in surface waters in coastal systems and the open ocean [21] where it can also contribute to primary productivity [20]. In the East China Sea, DON accounted for 44.0%–88.5% of the TDN, and urea and DFAA accounted for 1.7%–36.3% of the DON, before and during a *P. donghaiense* bloom [16]. In our experiments, *P. donghaiense* had similar maximum specific uptake rates for urea and amino acids under NO_3_^−^ -deplete and -replete conditions (Table 2) and half saturation constants for urea uptake were much lower than for NO_3_^−^, NH_4_^+^ and amino acids, suggesting a higher affinity for urea than for other N compounds tested. Further, during a bloom dominated by *P. donghaiense* in the East China Sea in 2005, maximum specific uptake rates for urea were 11–28×10^−3^ h^−1^ and half saturation constants were 0.25–14.95 μmol N L^−1^, which are comparable to results presented here for culture studies [16]. These results suggest that even if present at low concentrations, urea has the potential to be an important source of N fueling the growth of *P. donghaiense* and this may be important for out-competing co-occurring phytoplankton during bloom initiation and/or supporting the persistence of bloom organisms in the environment once NO_3_^−^ has become depleted.

A wide range of maximum uptake rates and half saturation constants for NO_3_^−^, NH_4_^+^, urea, and amino acid uptake have been observed among dinoflagellates both in laboratory cultures and experiments using natural populations during blooms (Table 4). Higher maximum uptake rates have been observed for NH_4_^+^ compared to NO_3_^−^, urea or amino acids for most species including: cultures of *P. minimum*, *Heterosigma akashiwo* and *Alexandrium minutum*, and assemblages dominated by *Dinophysis acuminate* (representing ∼91% of the phytoplankton biomass), *P. minimum* (representing >93% of the total phytoplankton cells), *Alexandrium catenella* (more than 99% of the phytoplankton) and *Karenia mikimotoi* (cell density up to 8×10^−6^ cell·L^−1^) (Table 4). In contrast, in cultures of the diatom *Pseudonitzschia australis*, maximum NO_3_^−^ uptake rates exceeded NH_4_^+^ uptake rates in populations growing on NO_3_^−^-deplete condition [54]. The high variability in maximum uptake rates and half saturation constants between species and experiments could be due to differences in the preconditioning of cells prior to kinetic studies. Nutritional history was a significant factor affecting N uptake rates by *P. minimum* culture [55]. N uptake kinetics are known to vary with in response to the physiological status and their nutrient history of cells [36]. Our culture experiments demonstrated that *P. donghaiense* can take up NH_4_^+^ and other N compounds at high rates even when ambient NO_3_^−^ concentrations are high (e.g., ∼43 μmol N L^−1^). This nutritional flexibility may contribute to the initiation and long duration of *P. donghaiense* blooms in nature. While blooms initiate when NO_3_^−^ concentrations are high, NO_3_^−^ is rapidly depleted as blooms progress.

**Table 4:** Summary of mean (±SD) maximum uptake rates (V_max_, ×10^−3^ h^−1^) and half saturation constants (k_s_, μmol N L^−1^) for ammonium (NH_4_^+^), nitrate (NO_3_^−^), urea and amino acid (glutamic acid (glu), glutamate (gln), glycine (gly) or amino acid mixture) uptake by HAB dinoflagellate species.

Species	Experiment type	NO_3_^−^		NH_4_^+^		urea		amino acids		Reference
		V_max_	k_s_	V_max_	k_s_	V_max_	k_s_	V_max_	k_s_	
*Heterosigma akashiwo*	Culture	18.0±1.08	1.47±0.25	28.0±2.17	1.44±0.35	2.89±0.24	0.42±0.16	-	-	[52]
*Prymnesium parvum*	Culture	-	-	-	-	-	-	10.5–13.1	0.09–0.10	[62] ^b^
*Prorocentrum minimum*	Culture	-	-	46–65	1.2–4.6	3E-01–8E-01	0.05–5.4	4–7	0.48–3.54	[63] ^c^
*Prorocentrum minimum*	Culture	95.8±5.5–341.2±90.7	0.68±0.23–23.3.0±11.1	1070±120.5–±88.7	2.48±0.8–6.23±0.47	149.2±12.3–480.5±13.4	0.86±0.16–1.82±0.59	271.6±35.6 517.1±125.8	14.1±6.8–22.6±9.9	[55] ^a, d^
*Prorocentrum minimum* (Choptank River)	Field	18.4±4.3–53.8±5.7	1.4±0.2–7.1±5.5	327±172.5–868.6±159	2.4±0.3–9.8±2.6	38.2±16.9–492.6±71.5	6.6±4.0–17.9±5.1	84.2±12.9–1516±134	4.8±2.3–26.6±4.1	[55] ^a, d^
*Prorocentrum donghaiense*	Culture (NO_3_^−^ deplete)	34.4±0.9	1.3±0.1	66.9±6.0	5.3±1.1	44.7±0.4	0.13±0.01	32.6±1.0	9.9±0.9	This study ^a^
*Prorocentrum donghaiense*	Culture (NO_3_^−^ replete)	-	-	74.7±3.4	7.1±0.4	39.7±0.2	0.12±0.01	31.4±0.4	12.5±0.1	This study ^a^
*Prorocentrum donghaiense* dominant	Field	-	-	700	3.9	17.0	5.3	25	1.8	[16] ^c^
*Karenia mikimotoi*	Field	18.0	43.6	500	4.9	20.0	1.4	11	1.7	[16] ^c^
*Alexandrium minutum*	Culture	0.29±0.01–0.70	0.22±0.02–0.28±0.06	0.65±0.01–1.49	0.25±0.04–0.38±0.04	-	-	-	-	[58] ^e^
*Alexandrium catenella*	Culture	3–47	0.6–28.1	26.0±2.0	2.0±0.6	25±8	28.4±15.0	-	-	[66]
*Alexandrium catenella*	Field	-	-	14.9±0.8	2.52±0.36	3.5±0.2	0.65±0.12	-	-	[39]
*Alexandrium catenella*	Field	24±2	4.6±1.1	64±5	8.4±1.7	61±8	43.9±8.8	-	-	[66]
*Dinophysis acuminata*	Field	3.5±0.2	0.79±0.26	13.9±0.2	0.67±0.06	6.2±0.6	0.53±0.22	-	-	[39]
*Lingulodinium polyedrum*	Field	22.4	0.47	47.1	0.59	61.6	0.99	-	-	[65]
*Cochlodinium* spp.	Field	0.9±0.0	1.0±0.4	-	-	1.9±0.1–2.2±0.3	1.6±0.2–6.6±2.0	-	-	[67]
Mixed dinoflagellate assemblages (Neuse Estuary)	Field	4.0±1.6	0.5±0.1	52.9±1.7	4.9±0.5	5.8±0.5	0.4±0.1	2.3±0.5	2.3±1.7	[55] ^a, d^

The ^a^, ^b^ and ^c^ indicate that an algal amino acid mixture, glutamic acid (glu) or glycine (gly) used as N substrates, respectively. The ^d^ and ^e f^ indicate the units are fmol N cell^−1^ h^−1^ and pmol N cell^−1^ h^−1^, respectively.

Studies have shown that nutrient preconditioning and physiological status [36], cell size [56], [57], growth rates [50], [55], [58], incubation time (minutes versus hours) [51], N substrate interactions [58]–[61], N preconditioning [53], [62], the DIN/DIP ratio [63], and environmental factors such as irradiance and temperature [59], [64], [65] can all contribute to variations in nutrient uptake kinetics. Because we conducted short incubations of uniform duration in culture systems acclimated to identical conditions, it is unlikely that these factors contributed to variability in uptake rates observed in this study.

While there are still few studies examining N uptake during blooms of *P. donghaiense*, kinetic parameters for NO_3_^−^, NH_4_^+^, urea, and glycine uptake were compared during successive dinoflagellate blooms in Changjiang River estuary and East China Sea coastal waters in 2005 [16]. In these mixed blooms, *Karenia mikimotoi* was the dominant species initially and then was succeeded by *P*. *donghaiense*. In most cases, when the bloom was dominated by *P*. *donghaiense*, the K_s_ values for urea and glycine uptake (K_s_, 5.3 and 1.8 μmol N L^−1^ respectively) were higher than when the bloom was dominated by *K*. *mikimotoi* (K_s_, 1.4 and 1.7 μmol N L^−1^ respectively) (Table. 4). The differences in the K_s_ of the two bloom species for NO_3_^−^ and NH_4_^+^ may have been a driver of species succession as concentrations of these compounds were drawn down during the *K. mikimotoi* bloom which preceded the *P. donghaiense* bloom.

Cell-normalized N uptake rates by *P. donghaiense*-dominated assemblages in the Chanjiang River estuary were comparable to cell-normalized N uptake rates measured in the culture studies reported here. Uptake rates of NH_4_^+^, urea, and glycine were ∼96, ∼22 and ∼38 fmol N cell^−1^ h^−1^, respectively, during blooms of *P. donghaiense* (cell density and Chl *a* were ∼5.0×10^6^ cells L^−1^ and 8.66–9.68 μg L^−1^, respectively) [11], [16] while maximum uptake rates for NH_4_^+^, urea and algal amino acids observed in this study were ∼119, 78 and ∼53 fmol N cell^−1^ h^−1^, respectively. This suggests that uptake may not have been saturated in the environment. The K_s_ values of NO_3_^−^ and urea for *P. donghaiense* were much lower than the environmental concentrations, but except for NH_4_^+^ and DFAA, which suggest that *P. donghaiense* have higher affinities on NO_3_^−^ and urea than those for NH_4_^+^ and DFAA, but the later two also contribute on *P. donghaiense* bloom initiation and duration.

## Summary

Results presented here demonstrate that *P. donghaiense* can grow on a diverse array of N compounds as their sole source of N, including inorganic N (NO_3_^−^ and NH_4_^+^) and organic N compounds such as urea, dissolved free amino acids, small peptides, and even cyanate to support their growth. Maximum specific growth rates varied by a factor of 2 for all of the N compounds tested. In addition, uptake kinetics for regenerated N sources (e.g., NH_4_^+^, urea, and amino acids) were similar under NO_3_^−^-replete and -deplete conditions suggesting that competition for these compounds may contribute to the success of *P. donghaiense* during bloom initiation when NO_3_^−^ concentrations are high, and maintenance, when NO_3_^−^ concentrations have been depleted. The nutritional flexibility exhibited by *P. donghaiense* likely contributes to its success in eutrophic environments where inorganic nutrient concentrations can be high, but where nutrient concentrations and the dominant form of bioavailable N rapidly change in response to stochastic events and the formation of algal blooms.
