# Correction: Growth and Nitrogen Uptake Kinetics in Cultured *Prorocentrum donghaiense*

**DOI:** 10.1371/journal.pone.0102767

**Published:** 2014-07-09

**Authors:** The PLOS ONE Staff

There is an error in the Funding statement for this article. The correct Funding statement is: “The authors acknowledge the financial support from the China Scholarship Council to Zhangxi Hu. In addition, this work was supported by NOAA and NSF grants to MRM, and Natural Science Foundation of China (NSFC) (Grant Numbers 40776078, 40876074, 41176104, U1133003). The funders had no role in study design, data collection and analysis, decision to publish, or preparation of the manuscript.”
